# Supplementary material for: Humidity response depends on the small soluble protein Obp59a in Drosophila
Source: eLife. 2018 Sep 19;7:e39249. doi: 10.7554/eLife.39249 (PMC6191283; doi:10.7554/eLife.39249)
Supplement: Supplementary file 1. — Cut sites were located 1-nt downstream (first large capital letter) of the 5’ end of the coding region, and 71-nt downstream (last large capital letter) of the 3’ end of the coding region, thereby removing essentially all of the coding sequence (blue letters) of Obp59a. Homology arms were used to replace the coding region with a DsRed marker. [file elife-39249-supp1.docx]

GTTGGCTTCGAAGGCAGGCGCTCGGCTGCGTCCTGGATGCAAGTGGAAAGTGGGAGGTGTGGGCGCCGCTGGGCGACGACCAGTCGGCTGGGCACTCTTCACCAGCTTGGGCTGGAGCTGCAGGCGCGACACTCTGTCCACGGACATGCTCTTGGGCCGCCGGGCTAGATAGCTATGGACGTGGCTATTGGAGCCATTGGCAATGTTCAAATTTACCAGGTAGTCCTGCAGCTGCGTCTTTAGCTGGCTGTTCTCCGCCTCCAGGCGACGCTTCTGTTGCTTCAGGCAGGCCACGTCCACGGCCACGTTGTTGACGCGTCGCCAGAAGGACTCCATTTGGGGTACCACTGCCACCTCGTCCTGGTACGTTAAACCAATGTTGAATGAAGAAATTACCTACTGGCATTTTTACTCACCTTAAGAAGATCAGTGGCTGGGTCAGTCAAGGAACCATTACCCTGATCCTCTATATCTCGAATCTCGATAATCTCCTTGGATATTTCCGGCAACTGGAACATCCGCTCCCGCTGGGTTTCCATCTTGGCACAGACCGCAGACAACTGTGCCAGAGTCTCGCCCTTGGCCACAGTTTCCTGGAGGAATTCCAAGGCATGGTAGCTCTCCACGGACATCAGCTTGAACAACTGGTGATCCCTGGCCACCTCCTGTTCCACACAGGTGCGTAGCTCCCTTAGTCGCTCGGTCAACTGCTGTTTGATGGCTTGCCGCTGCTCCAGTTGGCCCTGTTGCTTGATGTAGAACTCCTCGGAGACCAGACGCAAATTGGTCAGCTGGTCTACCAGGTGCTCGATTTCGTGGGTCTGCTGGCTAATCTGGAGGGCTGCCTCCTCATCCCGCTGCTTCAGATCCACGTACTCCGAGTAAAAGCCTTCGATGTGCTGACAGTAGCCCGCCAGAACACCCTGATACTCCTGCCACAGCTTCTCCAATCGAGCTTCACCTCTGGAGGTGATCTGCTCCAGCTGTAGTTGCATCTGGAAGTGGGGTTCAATTTAACATGTTTTCATAAACTTTTAAAATAACCTTTATTGTAAAATATAATCATCGAAGAGCATTGCACGCAATTGTAAAATTAAGATTGATATGTGTCTTGGTTCATTGTGCGTCAGTCACTCTGTAATTTGGATGCCATTCTCCAGAACTATAAATGATGTGGGCATAGCTGCTCCTCCATTCAGTTCCCAAGATGAAACAGTTGATTTTCCTGCTGATTTGTTTGAGCTGCGGCACCTGCTCCATTTACGCACTGAAATGCAGATCCCAGGAGGGACTAAGTGAAGCGGAGCTCAAGCGAACTGTGCGCAACTGTATGCATCGCCAGGACGAGGACGAAGATCGAGGACGAGGTGGACAGGGCCGGCAAGGAAATGGCTATGAGTACGGTTACGGAATGGATCACGATCAGGAGGAGCAGGACAGGAATCCAGGCAACAGGGGCGGCTATGGCAATCGAAGGCAGCGAGGACTAAGGCAATCGGATGGCAGGAACCACACCAGCAACGATGGAGGTCAGTGTGTGGCCCAGTGCTTTTTCGAGGAGATGAATATGgtaggtgatccaagagattcctttcaaagtcgaaatccttcagagaagcaggagcttattcctttcaattctcacccacagGTGGATGGCAATGGGATGCCCGATCGGCGCAAGGTGAGCTATTTGCTGACCAAGGACCTTCGGGACCGGGAGCTGCGCAACTTCTTCACGGACACCGTGCAGCAGTGCTTCCGCTATCTGGAGAGCAACGGAAGGGGCCGGCACCACAAGTGTTCAGCGGCCCGGGAACTGGTCAAGTGCATGTCGGAGTACGCGAAGGCGCAGTGCGAGGATTGGGAGGAGCACGGCAACATGCTCTTCAATTAGGATTAAACTCACCCCACTTTTTAGGTCCTCGCAGCGGTCCAAGAAACGCTCGTGGTTGTCCCGGATCCCGCCATCGGTGTTCTCCTCCAGCTGATAGAAGACGCACTCCAGTTGCTGCTGCTTCTGGGCATGCATCTCCTTGAACGCGGCGGAGTCCTGCTCCCACTGGCTCAGCACCGCCTGCTTGTCGTCCTCGTAGATCGTCCGAAAGAACTCGATGCGATCTCTATGGGTATCTGGGTAAAGGATAAGAAATAAATGGGTTAGAAATGAGGCCTAGTTGCGTTGAATGGTTTGTGTCCATTTTAATTTTCATTTTACAATGCTTCTTATTGTTCATAAAATGTACATCAAAATACTCCAGTAATTTTTGGATTTCCAAATATTTCAATGTTCTTATTAACGAAAGCATTAATATACATGTGAAATCCATTCCATTCATTTTAAGTATCTTTAAACATATTGCTGTAGCTGTGTATTTAGTTTCTGATTAAAATGTTATTTTTAAGGATGTTATCCCAATCGTACCAACTATCCTGGTGATATTCTCCATGTGTGACTGCTGCAGATTGGCGTACATATCCTCGGTGGATTCTATGTGAGCAATGAGGCGATGGATCACCTGGTCCTTGCGCTTAAGTGCCTCCTGAAAGAAAGACTCCACATCGACGATTTCGCGGCGCAGCTCCTGGATTTTCACCTGGCGCAAAATGCTCCGCCACTCCTGGTTGATTTTAGCCATATTGAGTCGACCAAAAGCATCCTCTCTTTTCAGTTTGTTCTATTTTAATGGGAAAAGTCATACCATAGGGATTTTGATTTTGATTTACTGCAAATTCGTGGTGTGTTACCTTCATGTACATGGAAATGAGCTGCATTTTCCGCCGCCTCGTCTCTTCCTCCATGTCGGCCCTCATTTGCAAATACCTGGCCCGCTCCTCCTCGGACATGTTGGCCAATTTGTTGCCTTTTCCTTTACCCTTTTTGCCCATGATTTAGTTTTTTTTTTTTACGGCTACCAAAAATCAGCTTATCAAATTTCAAAGGCCAATCTCTGACTAATTAGGCGAGCAAAGAGCCGCCTTGGAAAATAAATAATAAATTGACTGCCGGCGTTGCCATGACAAGATTCACTAGCCCACCCACTCCATATAATTTCGCTTTTCGTCGCCAGGTTTGTTGCCATAATAATGGATGCGAATTAAATTAATGCGAAACAGTGGAAAACGCCATTAGGGCTGTCCATCAAAGGGGAACCATTTGACATATAAAACTGAGGACGAACCGCTGGAAAGCTGTCATCATGCTGAGAATTGGTTTCGTGATTTGTGTGATTATATCACTGCGCTTGGTACGTACTC

Blue text = Coding sequence

Large capital letters = excised portion (replaced by DsRed cassette)

Green highlight = PAM sequences

Yellow highlight = Seed sequences

Red highlight = Remainder of seed sequences past Cas9 cut site

Teal highlight = Screening primers

Lowercase text = intron

Underlined text = homology arms
